# A community-based study on lower urinary tract symptoms in Malaysian males aged 40 years and above

**DOI:** 10.1038/s41598-022-05890-9

**Published:** 2022-02-11

**Authors:** Mohamad Fuad Mohamad Anuar, Muhammad Solihin Rezali, Mohamed Ashraf Mohamed Daud, Shaiful Bahari Ismail

**Affiliations:** 1 Sector for Biostatistic and Repository Data, Office of NIH Manager, National Institutes of Health, Ministry of Health Malaysia, Setia Alam, Selangor Malaysia; 2 Institute for Public Health, National Institutes of Health, Ministry of Health Malaysia, Setia Alam, Selangor Malaysia; 3 Urology Unit, Department of Surgery, Hospital Universiti Sains Malaysia, Health Campus, Universiti Sains Malaysia, Kubang Kerian, Kelantan Malaysia; 4 Department of Family Medicine, School of Medical Sciences, Universiti Sains Malaysia, Kubang Kerian, Kelantan Malaysia

**Keywords:** Prostate, Epidemiology

## Abstract

Lower urinary tract symptoms (LUTS) commonly affecting ageing men and is thought to be linked with other comorbidities and unhealthy lifestyles. This study was performed to report the prevalence of LUTS and its association with quality of life (QOL) in urination and other related factors. The study was part of the National Health and Morbidity Survey (NHMS) 2019, a cross-sectional community-based survey in Malaysia. Validated self-administered bilingual International Prostate Symptom Score (IPSS) was used to assess the LUTS. Other comorbidities and unhealthy lifestyles were recorded using face-to-face interview and in-situ measurements such as anthropometry assessment and blood measurement. There were a total of 2251 respondents. 16.3% of the respondents had clinically significant LUTS (IPSS ≥ 8). LUTS was found to be significantly associated with QOL, age and inactive physical activities. Nocturia was the most prevalent and bothersome symptom. LUTS is a common condition and adversely affect QOL. Ageing and physically inactive males are associated with the development of LUTS. It is recommended to increase public awareness of the condition and availability of treatment options for LUTS. Any upcoming survey should have a more in-depth investigation such as clinical profiling of subjects.

## Introduction

Lower urinary tract symptoms (LUTS) are a common problem and highly prevalent among ageing men^1,2^. The symptoms include a variety of urinary problems such as storage, voiding and post-micturition symptoms indicative of lower urinary tract dysfunction. The most common cause for LUTS in ageing men is benign prostatic hyperplasia or enlargement (BPH/E). However, there are also other causes for these symptoms, such as overactive bladder, chronic prostatitis and prostate carcinoma^3^. Previous studies stated that one in five men aged 40 and above suffer from moderate to severe LUTS (International Prostate Symptom Score (IPSS) ≥ 8)^3,4^.

The development of LUTS is associated with increasing age^3–8^, unhealthy lifestyles practices (such as physical inactivity, smoking habits and alcohol consumption)^3,9–11^ obesity^3,12^ and medical conditions (non-communicable diseases such as diabetes mellitus, hypertension and hyperlipidaemia)^3,8,12^. The presence of LUTS often results in poor quality of life (QOL), especially in urination and sleep^5^. Moreover, LUTS have also been shown to impact work productivity, libido, and emotional well-being negatively^3,13^. Despite the negative effects, many of them did not seek any medical advices for their symptoms^3,14,15^. The burden of the severity of LUTS, ageing, cultural differences, lack of symptom awareness and embarrassment factor are several reasons that influence the medical pursue among men^15–19^.

Previous studies on LUTS in Malaysia were conducted mainly in tertiary hospitals and a small, targeted population or patients^5,19–22^. To date, there is no population or community-based study done nationwide in Malaysia. Therefore, no reliable data for policy-makers to rely on to strategize proper health plans. With this limitation, we sought to provide more accurate relevant information, especially on men aged 40 years and above in Malaysia. Additionally, we also wanted to relate any association between LUTS and QOL due to urinary symptoms and to delineate between the clinical and lifestyle factors associated with LUTS.

## Results

### Prevalence of LUTS and its related factors

Overall, 16.3% (95% CI: 14.1, 18.8) of men aged 40 years and above were considered to have LUTS. A higher prevalence was observed in the elderly age group (60 years and above) with 23.9% (95% CI: 19.8, 28.6), an increment of 13% from aged 40 to 49 years. Based on the unhealthy lifestyles, the highest prevalence was among physically inactive men with 19.9% (95% CI: 15.5, 25.2). Among the disease-related LUTS, hypertension had a higher prevalence of 19.5% (95% CI: 16.5, 22.9) (Table 1).

**Table 1:** Prevalence of LUTS by demographic, risky lifestyles and disease related in Malaysia.

Demographic	N	Prevalence (%)
**LUTS**	396	16.3 (14.1,18.8)
**Strata**		
Urban	242	16.2 (13.6,18.8)
Rural	154	16.8 (13.6, 20.6)
**Age (years)**		
40–49	81	10.9 (8.1, 14.5)
50–59	117	16.3 (13.0, 20.2)
≥ 60	198	23.9 (19.8, 28.6)
**Ethnicity**		
Malay (including indigenous)	250	16.1 (13.8, 18.8)
Chinese	63	14.5 (9.9, 20.7)
Indian	21	15.6 (8.9, 26.0)
Borneo	46	21.5 (14.9, 30.0)
Others	16	18.5 (8.8, 34.7)
**Risky lifestyles and disease related**		
Smoking	180	15.3 (12.5, 18.7)
Alcohol user	40	14.4 (9.4, 21.4)
Overweight (BMI ≥ 25.00)	192	15.7 (13.2, 18.6)
Abdominal obesity (WC ≥ 90.0 cm)	194	16.1 (13.5, 19.0)
Physical inactive	126	19.9 (15.5, 25.2)
Hypertension	224	19.5 (16.5, 22.9)
Diabetes mellitus	145	17.4 (14.3, 21.1)
Hypercholesterolemia	201	16.1 (13.5, 19.0)

**Figure 1:**
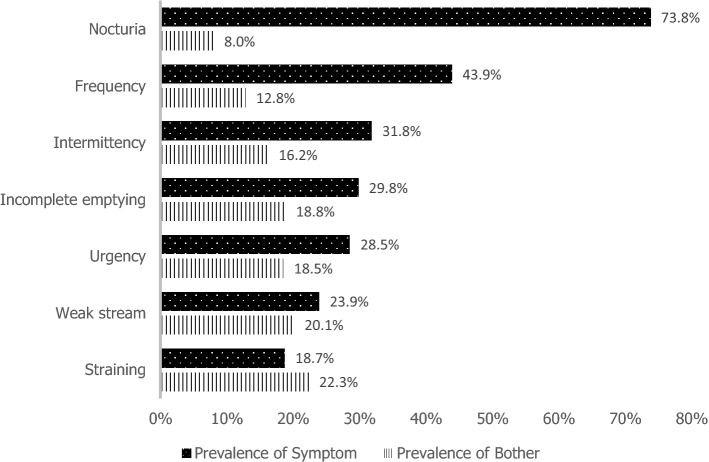
Prevalence of lower urinary tract symptoms (LUTS) and bother.

By symptom, the most prevalent symptoms among respondents were nocturia with 73.8%, followed by frequency (43.9%) (Fig. 1). In terms of “bothersome symptom”, higher prevalence was in straining symptom with 22.3%, followed by weak stream, 20.1% (Fig. 1). The ‘bothersome’ of each symptom was closely associated with the bivariate analysis showing p ≤ 0.05 for all symptoms in relation to their ‘bothersome’.

### Severity of LUTS and quality of life in urination

In our study population, 353 (15.7%) had no LUTS, 1502 (66.7%) had mild LUTS, 339 (15.1%) had moderate LUTS and 57 (2.5%) had severe LUTS. There was an increase in prevalence of clinically significant (moderate to severe, IPSS score ≥ 8) LUTS with age—11.8% in respondents aged 40 to 49 years old towards 24.9% in ≥ 70 years old (about 13% increment). Higher prevalence of low QOL in urination was seen in clinically significant LUTS with 73.7% (Table 2). Moderate correlation was found between symptoms score and QOL in urination score (r_s_ = 0.558, p-value ≤ 0.001).

**Table 2:** Severity of LUTS stratified by age groups and poor quality of life (QoL).

Severity of LUTS (by IPSS scores)	40–49	50–59	60–69	> 70	Poor QOL
Asymptomatic (0)	126 (18.3%)	121 (16.6%)	71 (13.5%)	35 (11.2%)	2 (1.1%)
Mild (1–7)	480 (69.9%)	489 (67.3%)	333 (63.5%)	200 (63.9%)	45 (25.1%)
Moderate (8–19)	75 (10.9%)	98 (13.5%)	102 (19.5%)	64 (20.4%)	95 (53.1%)
Severe (20–35)	6 (0.9%)	19 (2.6%)	18 (3.4%)	14 (4.5%)	37 (20.7%)
Total	687 (100%)	727 (100%)	524 (100%)	313 (100%)	179 (100%)
Clinically insignificant (0–7)	606 (88.2%)	610 (83.9%)	404 (77.1%)	235 (75.1%)	47 (26.3%)
Clinically significant (8–35)	81 (11.8%)	117 (16.1%)	120 (22.9%)	78 (24.9%)	132 (73.7%)
Total	687 (100%)	727 (100%)	524 (100%)	313 (100%)	179 (100%)

### Factor associated with LUTS

For factors associated with clinically significant LUTS (after adjusting with demographic, lifestyles and related diseases), increased age was significantly associated with LUTS with aOR—men aged 50 to 59 years old; aOR 1.49 (95% CI: 1.09, 2.03), men aged 60 to 69 years old; aOR 2.22 (95% CI: 1.62, 3.04) and men aged 70 years and above; aOR 2.32 (95% CI: 1.62, 3.33). Inactive men were significantly associated with clinically significant LUTS with AOR of 1.57 (95% CI: 1.23, 2.00). Other factors were found not significant in this study.

## Discussion

The findings from this study have provided a better view of the prevalence of LUTS and the associated factors in our population. The prevalence of LUTS in Malaysia for all men aged 40 years old and above was 16.3%, and increased by 13% towards the elderly age group (60 years and above). The prevalence was low as compared to other studies conducted in Malaysia^5,19–22^. These studies focused on small population in a certain locality or targeted individuals such as outpatients who sought medical treatment in tertiary hospital. Comparatively, the prevalence of LUTS was almost similar with findings from Singapore^3^. However, in a recent review study done in Asian countries demonstrated that the prevalence of LUTS was higher than our findings^25^. The study also quoted higher prevalence by age groups than our study. However, few other studies performed in developed countries such as USA and Netherlands showed lower prevalence of LUTS^26,27^. Several reasons may contribute to the differences, such as the proportion of elderly persons, male gender population ratio and methodological approaches.

Our study had shown that the severity of LUTS commonly affects the QOL among men. This correlation finding was also documented by other investigators^3,9,28^. From our study population, nocturia is the most prevalent symptom, followed by frequency, intermittency and incomplete emptying. Similarly, previous reports described nocturia to be the most prevalent symptom in men having LUTS^3,29,30^. Nocturia symptom greatly effect on sleeping time which can cause adverse medical mental dysfunction. Moreover, this symptom can lead to unhealthy physical health and poor QOL^31^. Knowing the most prevalent symptom will allow healthcare providers to offer various interventions and medical advice at an earlier stage. This will ensure better overall QOL of the patients and avoids any disease-related condition due to long term-effect of the symptoms.

For factors associated with LUTS, our study had found that increasing age and lack of physical activity were significantly related. We did not find any association between the following factors: sociodemographic, obesity, abdominal obesity, hypertension, hyperlipidaemia, diabetes mellitus, and risky lifestyles (smoking and alcohol intake), with LUTS. As reported, increasing age commonly led to LUTS emergence among men aged 40 years old and above^1,3–8,25,30,32–35^. Clinical progression of prostate enlargement may be the cause for LUTS after the age increases^30,35^. Good physical activities adversely reduce the progression of LUTS. Moreover, being physically active may also reduce other chronic diseases or conditions^33^.

Ministry of Health (MOH) Malaysia had started to show a keen interest in men’s health since 2002, with National Men’s Health Plan of Action Malaysia 2018–2023 was introduced in 2019^36^. Currently, age friendly men’s health clinics which focus on male-specific conditions were established to provide primary health care among the men population aged 40 years and above. Besides, the clinic also provided consultations or medical advices on men’s health amongst younger age group. The clinics were under supervision of MOH Malaysia and all standard operating procedures and regulations were followed the current guidelines provided by the MOH. Presently, the clinics are free and open to all public especially for male. In addition, health screening among men in the community or workplace was also being carried out and increases of male clinic attendances was done to improve the availability and accessibility of health services to men.

With the establishment of men’s health clinic, MOH Malaysia had started focus on human resource development by prioritize on building capacity in providing men’s health services. The development and conduct training on men’s health modules towards healthcare professionals was regularly done. The modules comprised of management of men’s health issues such as LUTS and also healthcare aspect on the sexual reproductive health. Health information system on men’s health status was also established for the purpose of disseminating and reporting on men’s health statistics as well as documentation on patient’s information and cases. Furthermore, clearing house concept was introduced to aid researchers and stakeholders to store and receive information regarding the men’s health issue in Malaysia.

The findings of this study will help the stakeholders to support the establishment or even giving additional funding for the service to progress. Furthermore, the findings will also initiate the development of novel strategies and treatment towards LUTS. The scopes of the development comprised of comorbidities and risky lifestyles screening, screening tools for men’s health, personal management of out-of-pocket expenditure and healthcare utilization. In addition, further guideline needs to be added for moderate/severe case of LUTS to investigate for possibility of prostate cancer. Research collaboration on LUTS and other men’s health issues should also be made with other institutions and universities.

We acknowledge that this study has several limitations. The cross-sectional nature of the study does not allow the establishment of a temporal relationship of LUTS with the factors, allowing only association with relevant factors during the particular time of duration when this survey was conducted. The survey concept may lead to biases and precisions due to the unwillingness of the participated men to answer the instrument sincerely.

In summary, the findings of this study showed that LUTS are fairly common in men aged 40 years and above amongst Malaysian population, with age and lack of physical activities were associated with the LUTS conditions. QOL moderately affected a large proportion of individuals with LUTS. As LUTS adversely affect individual’s QOL, increasing public awareness and availability of treatment options are needed to address this problem facing the growing number of elderly men in the population.

## Materials and methods

### Survey design and data collection

This study was part of the National Health and Morbidity Survey (NHMS) 2019: Non-Communicable Diseases, a cross-sectional survey that implemented a national complex survey study design covering the whole urban and rural Malaysia (13 states and 3 federal territories). The sampling frame consists of selected enumeration blocks (EBs) same as that was provided by the Department of Statistics Malaysia in NHMS 2015. To ensure the national representativeness, two stage stratified random sampling was used. The primary stratum will be the states and federal territories and the secondary stratum will be the urban and rural strata formed within the primary stratum. The sample size was calculated using a single proportion formula based on NHMS 2015 which derived 5,676 LQs. The sampling procedure consist of two stages which were the primary sampling unit (PSU) for the EBs selection and the secondary sampling unit (SSU) for living quarters (LQs) within the EBs. 475 EBs were selected with 362 EBs from urban areas and other 113 EBs from rural areas respectively. Twelve LQs were randomly selected within the selected EBs. All individuals residing for at least two weeks in the LQs prior to data collection were eligible to participate in this survey. Details on the methodological survey can be seen elsewhere^23^. All procedures had been approved and obtained the ethical approval from the Medical Research and Ethics Committee of the Ministry of Health Malaysia. This survey was registered at the National Medical Research Register (NMRR), bearing the number of NMRR-18–3085-44,207. The survey was conducted according to Ministry of Health Malaysia guidelines and regulations to ensure that the ethical was abide during the data collection. The data collection was initiated from July 2019 to October 2019, with a training course for data collectors, nurses and field supervisors conducted in July 2019. All the participants were provided with a bilingual informed consent form (Malay and English) which stated the purpose of the survey and methods used. All the informed consents were obtained before the study conducted. A total of 2,251 men aged 40 years and above were recruited for this study, after excluding of those with significant cognitive impairment, speech deficit impairing communication or illiterate (cannot read) and incomplete survey data.

### Survey instrument

A structured questionnaire comprised of sociodemographic and lifestyles were used to collect the data and was based on previous NHMS 2015^23^. The questionnaires were bilingual (Malay and English), assuming all the Malaysian fluently in either languages. Clinical assessments such as clinical blood measurement (fasting blood glucose, cholesterol level, blood pressure) and anthropometry measurement were done by trained nurses. Lifestyles practices such as physical activity, smoking and alcohol intake were assessed, to evaluate risky lifestyles practices among Malaysians. LUTS was assessed and measured using International Prostate Symptom Score (IPSS), a well-established diagnostic screening tool adapted into Malay version^24^. The IPSS was based on the seven questions concerning urinary symptoms and one question regarding the QOL of the urination. Each answer for urinary symptoms was assigned into points from 0 to 5 (0 = not at all, 1 = less than 1 in 5 times, 2 = less than half the time, 3 = about half the times, 4 = more than half the times, 5 = almost always). The total score, therefore, ranges from 0 to 35. Based on the score, respondents were classified into asymptomatic (0 points), or having mild LUTS (1–7 points), moderate LUTS (8–19 points) or severe LUTS (20–35 points)^5,22,24^. Clinically insignificant LUTS was defined based on the score 0 to 7 while clinically significant LUTS, 8 to 35 points^3^. The QOL for urination was assessed on question 8 and can be divided into good (Score 0–1) and poor (Score 2–6)^22^. The selection and score of the QOL as follow; 0 = delighted, 1 = pleased, 2 = mostly satisfied, 3 = mixed feeling, 4 = mostly dissatisfied, 5 = unhappy, 6 = terrible. For bother aspect, the factor should be assessed by using the question of the QOL. To quantify the bother, score 0 to 2 were grouped as ‘satisfied’, score 3 was ‘equally satisfied and dissatisfied’ and score 4 to 6 were grouped as ‘dissatisfied’. The bother aspect was defined as the respondents ‘dissatisfied’ with their symptoms^3^.

### Statistical analysis

Data analysis was conducted using IBM SPSS for Windows, Version 21.0. Univariate analysis was performed to examine the distribution of sociodemographic factors, symptom severity, quality of life, risky lifestyles and disease-related of the study population. Complex sample analysis was done to illustrate the prevalence of LUTS by sociodemographic and other variables. The weighting factor was applied to adjust for non-response and for the varying probabilities of selection. The detailed calculation for weighting factor was stated in NHMS 2019^23^. The output will be representative of Malaysian men aged 40 years and above. Spearman’s test was done to compare symptoms score and QOL score with a significant level of p < 0.05. Factors associated with LUTS were determined at both univariate and multivariate levels by using logistic regression analysis. The outcome was a binary variable coded as “0” for clinically insignificant and “1” for clinically significant for LUTS. Variable selection was done using the forward and backward stepwise logistic regression method. The final model was presented with adjusted odd ratio (aOR) and 95% CI, Wald-statistics and P-value, with the level of significance was set at p-value less than 0.05. Multicollinearity and interaction were checked with classification table and receiver operating characteristic (ROC) curve was done to ensure the quality of the model was acceptable.

## Data Availability

Data available upon request with reason.
